# Association for Human Pharmacology in the Pharmaceutical Industry conference 2022: impending change, innovations and future challenges

**DOI:** 10.3389/fphar.2023.1219591

**Published:** 2023-11-02

**Authors:** Charles Mundy, James Bush, Joseph Cheriyan, Ulrike Lorch, Steffan Stringer, Jörg Taubel, Kirsty Wydenbach, Timothy C. Hardman

**Affiliations:** ^1^ Niche Science & Technology Ltd., Richmond, United Kingdom; ^2^ Covance Clinical Research Unit Ltd., Leeds, United Kingdom; ^3^ Cambridge University Hospitals NHS Foundation Trust, Cambridge, United Kingdom; ^4^ Richmond Pharmacology Ltd., London, United Kingdom; ^5^ Alwyn Consulting, Guildford, United Kingdom; ^6^ Weatherden Ltd., London, United Kingdom

**Keywords:** early phase clinical development, meeting report, association for human pharmacology in the pharmaceutical industry, drug development, innovation

## Abstract

The Association for Human Pharmacology in the Pharmaceutical Industry’s annual meeting focused on current and impending challenges facing the United Kingdom’s (UK) pharmaceutical industry and how these opportunities can inspire innovation and best practice. The UK pharmaceutical landscape is still evolving following Brexit and learnings from the coronavirus disease 2019 (COVID-19) pandemic. As such, the UK’s clinical community is in a unique position to steer innovation in a meaningful direction. With the continuation of remote forms of working, further opportunities have arisen to support novel practices away from the clinic. The keynote speaker reflected on clinical development over the past 40 years and how the industry must continue to concentrate on patient welfare. The future of drug development was discussed regarding challenges associated with developing translational gene therapies, and the status of investment markets analyzed from a business strategy and consulting perspective. The patient viewpoint was a core theme throughout the conference with patient-centric blood sampling and decentralized clinical trials providing suggestions for how the industry can save costs and increase efficiency. Moreover, the patient perspective was central to a debate over whether ethics requirements should be the same for oncology patients taking part in first-in-human studies as those for healthy subjects. Discussions continued around the changing roles of the Qualified Person and Principal Investigators which underpins how sponsors may want to run future trials in the UK. Lessons learned from conducting challenge trials in healthy volunteers and patients were discussed following a presentation from the serving Chair of the COVID-19 challenge ethics committee. The current state of interactions with the Medicines and Healthcare products Regulatory Agency were also explored. It was considered how the immediate future for the UK clinical trials community is inevitably still linked with Europe; the newly implemented European Medicines Agency Clinical Trials Information System has been met with lukewarm responses, providing a promising opportunity to ensure UK Phase I units continue to play a vital role in global research.

## Introduction

Established in 1988, the Association for Human Pharmacology in the Pharmaceutical Industry (AHPPI) is a not-for-profit organization that provides a forum for continuing education in clinical pharmacology and fosters discussion regarding all issues around early phase drug development and clinical trials. The AHPPI’s annual 1-day meeting, held in London on 7 December 2022, brought together stakeholders from a broad range of backgrounds to discuss how current and impending challenges in early phase drug development are inspiring future innovations in the pharmaceutical industry. This conference facilitated discussion between a broad range of industry professionals. This article is a conference review and data are based on literature as well as personal experience.

## Morning session

The conference opened with an introduction from the AHPPI Chairman, Dr. Tim Hardman, who provided an oversight on the shifting pharmaceutical and biotechnology landscapes. The coronavirus disease 2019 (COVID-19) pandemic has acted as a pivot point for evolving pharmaceutical markets and necessitated new approaches to the drug development process. The pandemic highlighted the industry’s resilience and creativity that were reflected in its ability to bring both vaccines and COVID-19 therapeutics online despite moving towards remote working. Focus is shifting away from traditional in-person clinic visits. Innovative solutions and collaborations such as remote blood sampling and decentralized clinical trials perpetuate the debate as to whether all investigations need to be conducted in a clinical setting and the arising opportunities for remote forms of research.

Dr. Hardman discussed the future direction of clinical developments in the United Kingdom (UK). He commented on how the repercussions of Brexit on the UK pharmaceutical industry have yet to be fully expanded, and the full consequences of the Medicines and Healthcare products Regulatory Agency’s (MHRA) separation from the European Medicines Agency (EMA) is not currently clear. Strategies are encouraged whereby the UK can take advantage of any opportunities that may arise from the introduction of the new European Union (EU) Clinical Trials Regulation and the Clinical Trials Information System (Clinical Trials Regulation, 2014; Clinical Trials Information System, 2023).

Future prospects should also be explored through the UK’s leading pharmaceutical industries. According to the Association of the British Pharmaceutical Industry’s 2021 report, oncology is the UK’s strongest area of clinical research (Association of the British Pharmaceutical Industry, 2021). The broad field of cell and gene therapy promises a number of innovative treatments that are likely to become important in preventing deaths from cancer. Despite these promising opportunities, there are still significant challenges to be faced, which emphasizes the underlying uncertainties for the future generations of clinical and regulatory professionals, and how they will be trained. Moreover, questions remain about how the pharmaceutical industry will adapt to the shift in focus from symptomatic resolutions towards prevention and cure. Thirty-four years on from the first AHPPI conference the regulatory hurdles are higher, the timelines longer, and costs greater.

## Challenges of bringing a gene therapy to the clinic

Dr. Piv Sagoo (Orchard Therapeutics) directed the opening presentation speaking about the difficulties faced in bringing cell and gene therapies from preclinical research through to the clinic. The presentation focused on Orchard’s approach and considerations to hematopoietic stem cell gene therapy, and finished with a case study concerning Libmeldy™, a drug designed to treat children with metachromatic leukodystrophy (MLD) (European Medicines Agency, 2020).

**FIGURE 1:**
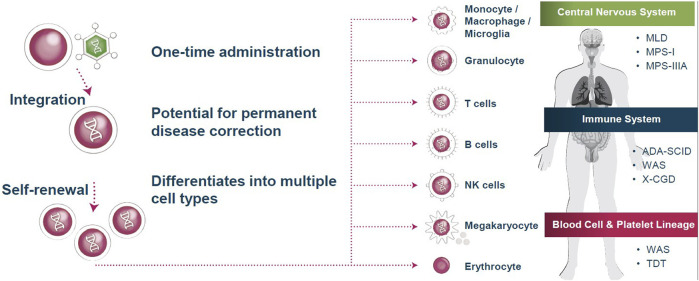
Current approaches to treating rare genetic diseases exploit stem cell lineage differentiation. By integrating gene therapy into hematopoietic stem cells, self-renewing and differentiating progeny produce the therapeutic molecule or protein in a range of immune cell subsets. These altered immune cells are then utilized to treat a variety of diseases throughout the body. MLD = metachromatic leukodystrophy; WAS = Wiskott-Aldrich syndrome; NK = natural killer; TDT = transfusion-dependent β-thalassemia; ADA-SCID = adenosine deaminase severe combined immunodeficiency; X-CGD = X-linked chronic granulomatous disease; MPS-I = mucopolysaccharidosis type I; MPS-IIIA = mucopolysaccharidosis type IIIA. Source: Orchard Therapeutics.

Orchard Therapeutics use an *ex vivo*, autologous, hematopoietic stem cell gene therapy approach to treating rare genetic disorders. A patient’s own hematopoietic, blood, or bone marrow stem cells are extracted and genetically transduced with a self-inactivating HIV-based lentiviral vector. Once modified, the patient’s stem cells can be cryopreserved to be re-introduced intravenously once they have recovered from the initial apheresis procedure. Patients undergo a degree of myeloablative conditioning tailored to their specific profile. Once the modified cells are integrated stably within their bone marrow ‘niche’, the progeny can produce the therapeutic molecule or protein in a range of immune cell subsets (Figure 1). This gene therapy approach allows researchers to deliver targeted gene expression to different cellular subsets where needed, or induce constitutive expression within all cells. Thus, therapeutic gene expression can theoretically be targeted to address a range of disease indications across different physiological systems. Dr. Sagoo recounted how Orchard Therapeutics’ current pipeline is heavily focused on early phase preclinical research within the neurogenerative and neurometabolic arenas, both of which are considered competitive spaces in the gene therapy research space.

Working with a gene therapy will most likely require a more progressive regulatory pathway than is currently available. The drug development process for gene therapies is viewed very differently by regulatory authorities to accommodate this broad field with vastly different therapeutic approaches. When also working in a rare disease setting, there is a greater propensity towards taking a risk-based approach to drug development. High qualities and standards still apply to the traditional aspects of drug development, such as study design and conduct, relevance of animal models, pharmacology, toxicology, and route of administration. However, regulatory authorities often demand more stringent proof-of-concept requirements, with a focus on the safety and efficacy of manufacturing quality. In addition, cell isolation, viral production, cell modifications, and biodistribution have to be faultlessly defined.

The unique complexities involved in bringing a gene therapy through the drug development process often requires a different approach to study structure. Although traditional studies often include large patient cohorts, diseases targeted by gene therapies often have a smaller pool of patients to recruit from, maybe even only one or two. As such, agencies allow some flexibility in how these trials are designed. Therapies often require several multi-step procedures, such as apheresis requiring stem cell mobilization, the apheresis itself, and immune conditioning which, in itself, has numerous associated risks and adverse effects. To aid with these complexities, regulatory authorities provide guidance for sponsors on such aspects as the manufacturing process, applicability of data packages, criteria for selecting a therapeutic vector, or for the suitability of *in vivo* or *in vitro* data or models being presented.

One aspect of cell and gene therapy that is of particular interest to both pharmaceutical companies and regulatory authorities focuses on dosage selection. Determining the dose of a gene therapy impacts several aspects of the medicinal output. Essentially, dosage relates to the level of transgene expression, which is directly linked to the number of copies of the gene that has been integrated (referred to as vector copy number). Dosage also affects the degree of a gene product’s chimerism. Equally, lentiviral integration must have no potential for oncogenicity. Considering how dosage affects the performance of the drug product, regulatory authorities require sponsors to provide impeccable data. One suggestion that was discussed was the potential for a ‘platform’ approach to harmonize development and minimize concerns. It was agreed that a platform approach could ease the regulatory process for rare diseases and reduce time and effort spent on debating issues relating to lentiviral vector production, cell isolation, and medicinal product manufacturing.

Dr. Sagoo detailed the success story behind Orchard Therapeutics’ Libmeldy™, a drug designed to treat MLD. This neurodegenerative disease exhibits progressive symptoms of cognitive and motor decline, and ultimately leads to death (National Institute of Neurological Disorders and Stroke, 2023). The clinical challenge for therapies often sits with the fact that diagnosing symptomatic patients often means that patients are already suffering the long-term consequences of the disease. For example, patients with MLD already have extensive levels of neurodegeneration on diagnosis. Patients should ideally be diagnosed and treated before any symptoms of MLD manifest. This is most commonly achieved by family screening of current MLD patients to identify those at risk.

In concluding the presentation, Dr. Sagoo noted that Orchard Therapeutics has success in developing drugs that prevent neurodegeneration (Fumagalli et al., 2022). In addition to these successful medicinal products, they have been working with regulatory authorities to accelerate the clinical pathway within this innovative and rapidly growing field. The focus now turns to ensuring the suitability of the chemistry, manufacturing, and controls package of gene therapies, enabling a platform approach to simplify regulations in the future.

### Debate: the ethics requirements for conducting phase I trials should be the same in oncology patients as healthy subjects

For the Motion: Dr. Ayad Abdul-Ahad (Niche Science & Technology);

#### Against: Dr. Stephanie Ellis (Health Research Authority)

Phase I clinical trials aim to verify that an investigational medicinal product (IMP) is safe to use in humans. These early phase studies aim to recruit healthy volunteers as a means to determine the safety of new medicine candidates. This well-established means of testing has adopted a firm set of ethical standards to ensure these investigations minimize the risk to the volunteer. An exception to this development pathway is in the field of oncology. Despite vast improvements in cancer treatments, more than 10 million people died of cancer worldwide in 2020 [www.WHO.int]. It is clear that oncology is a unique field for clinical studies. Due to the difficult situation that many patients face, Phase I oncology studies appear to offer hope of some respite and recruit those diagnosed with cancer to assess and maximize any benefits the drug may have for the patient. As such, patients and physicians are often prepared to accept a more lenient risk-benefit profile. However, debate has arisen as to whether the ethics requirements for conducting Phase I trials should be equal across oncology studies as those for healthy volunteers.

Supporting the motion was Dr. Ayad Abdul-Ahad (Niche Science & Technology). Dr. Abdul-Ahad is an immune-oncologist who has led pharmaceutical global clinical development, medical affairs, and regulatory strategy for more than 30 years. Dr. Abdul-Ahad addressed the two primary arguments criticizing the conduct of Phase I oncology studies: The poor risk-benefit ratio and the lack of appropriate informed consent.

Phase I oncology studies have what is well known to be a poor risk-benefit ratio, with only 2.7% of patients experiencing a complete response (Chihara et al., 2022). However, in patients where standard medicinal interventions have failed, a slight chance of therapeutic benefit may be favorable. Dr. Abdul-Ahad maintained that the risk-benefit ratio should always be considered within the context of available alternatives. Furthermore, he noted that benefits to cancer patients do not solely lie in improved response rate, rather an absence of infirmity in addition to the importance of psychological and social dimensions. Regular physician contact can reduce psychological burden and some patients in Phase I studies experience a better quality of life than those only receiving supportive care. Ultimately, improving quality of life is a benefit that must never be ignored.

Critics of early phase oncology studies note that cancer patients are not always in the ‘correct’ mindset to provide informed consent. However, most of the people who make these claims are themselves healthy and cannot truly sympathize with patients. Those who volunteer for oncology trials often have a differing opinion on what risks are considered unfavorable, where for them, all other therapies tended to have failed. The lengths that palliative patients are prepared to traverse and the challenges they are prepared to endure are considerable, simply because it gives them hope. Many patients consent knowing that it is unlikely the trial will benefit themselves, but take part for the promise it offers to future patients, providing some with meaning in their final days.

Overall, the new model of cancer therapies is designed to benefit patients in more ways than improved responses to tumor growth and progression. Gone are the days when doctors treat patients by trying to ‘kill’ the tumor before the individual. New Phase I protocols are tailored to the specific pathophysiology of different cancers and select the appropriate patient populations most likely to benefit from the new therapy in the era of personalized medicine.

Countering the motion was Dr. Stephanie Ellis (Health Research Authority). Dr. Ellis adjusted her argument to emphasize that individuals involved in Phase I oncology trials need to be better informed before agreeing to take part in a study. Dr. Ellis argued it is unethical for an individual to provide consent when it is difficult under current models for volunteers to be appropriately informed of the risks.

Dr. Ellis has over 30 years of experience both participating in and chairing research ethics committees. She recounted how during this time, a recurring theme that led her to counter the motion is that patient information leaflets are often not suitably tailored to the target individual, with complex wording and lengthy documents. The response to increased trial complexity has been simply to provide more and more information. Often, superfluously long and indigestible information sheets are provided to patients, leaving them unsure of what is actually required of them. All participants, especially oncology patients, face an unexpected and often daunting dilemma when considering whether or not they should participate in a clinical trial. Information sheets should be accommodating of this situation, presenting content in a concise and easily understandable manner. Although the need for standards within the regulatory industry is paramount, the fact that information sheets require the same level of scrutiny may be misplaced. Medical writers produce information sheets to address these regulatory requirements, yet sometimes the needs of the target audience get lost in the process. Dr. Ellis argued that it is important for clinical professionals to acknowledge the viewpoints of those who do not share the same mindset of a healthy society.

The method of how a patient should be presented with the necessary information is debated among many research ethics committees. Although leaflets have been the resource used for decades, technology may provide ways to revolutionize how information is presented to participants. Ultimately, the sole purpose of an information sheet is for the reader to understand the study and how it may affect them. Dr. Ellis recognized that some members of ethics committees are resistant to change, and many are insistent on still using the current information sheet format. This disconnect between what research ethics committees want and what participants need must be addressed before any updates can be adopted.

Following both talks, the speakers found common ground for both arguments. Overall, it was acknowledged that oncology trials are invaluable, the contribution made by the patients incalculable, and that patient information sheets need to be better adjusted to inform the lay individual.

### The role of the Qualified Person in post-Brexit Britain

Pam Turner (PNR Pharma) discussed how her role as a Qualified Person (QP) in the UK has changed since the Brexit referendum. Ms. Turner became a British QP in 2004 and also received her EU QP certification in Ireland as a way of staying competitive post-Brexit. Following the UK’s departure from the EU, the MHRA’s approach to clinical trials has diverged from the EU regulation through numerous annexes. Annex 21 concerns the import and export of medicinal products (European Commission, 2022). These changes have impacted noticeably on the role of the QP in the United Kingdom.

Annex 21 was introduced in August 2022 and implements legislation for the import and export of medicinal products from outside the EU and European Economic Area (EEA). A focal aspect of Annex 21 is that fiscal transactions are not applicable, QP certification only takes place after physical importation and customs clearance of the imported products. Sites with specific importation responsibilities include the site of physical importation and the site of QP certification—this has been a point of contention considering clinical trial sites have traditionally remained clear of QP involvement. Annex 21 also requires the maintenance of extensive documentation for drugs imported into the UK, and checking this documentation is another responsibility of the QP. This verification covers details regarding ordering and delivery, the dispatch site, the destination site, shipping details, and full customs documentation. Further checks must also ensure that any third country manufacturing sites have the same retention policy as the EU. Full batch documentation must also be covered as a new aspect of the QP certification. If the product has subdivided batches, these multiple batches, as well as the whole batch, must be reconciled. Furthermore, a QP declaration is required to cover all activities performed for UK trials outside the EU, EEA, and United Kingdom. For studies in the EU that include any activities in the UK, the QP declaration must also cover the UK portion as well. Another change imposed by Annex 21 is that equivalence now needs to be shown to the EU. Overall, the process of importing an IMP from outside the EU or EEA is extensive and complex, and introduces several new responsibilities to the role of the QP.

To import a product from within the EU or EEA, QPs now have to perform a QP oversight, with the MHRA taking full legal responsibility for the importation. For a UK QP to provide a Manufacturer’s Authorization for an IMP, the product must be fully labelled for availability in the EU and verified by a European QP. The product can only be shipped to an appropriate UK trial site, with sufficient quarantine systems, that was named in the UK clinical trial authorization (CTA) application. Necessary documentation for importation includes the following: A UK CTA application; evidence that the certifying site is in a listed EU country and has the appropriate IMP for the dosage form; certification for associated activities such as manufacturing, packaging, and testing; an approved UK trial site from an ethics application; shipments and exclusions; and a written agreement for importation between the sponsor and European QP. Equally complex verification processes are also in place for batch certifications, importation of Non-IMPs, auxiliary medicines, unmodified comparators, commercial products, and imports into Northern Ireland. When entering Northern Ireland, IMPs can be certified via the UK or the EU. Ms. Turner stressed that the process to import IMPs from the EU used to be significantly simpler and easier, and this added complexity is likely the primary reason for the lack of new QPs being trained in the United Kingdom.

Another direction where the MHRA has diverged from the EMA regards Annex 16, which applies to all UK studies that require full QP certification (European Commission, 2015). Since Brexit, trials no longer need to comply with EU Directive 2001/20 or the Clinical Trials Regulation following its implementation. Instead, UK trials adhere to the 2004 Humans Medicines Act and Regulations (amended by the Brexit regulation [2019]) (UK Government legislation, 2004), as does QP certification.

In conclusion, Ms. Turner admitted that the role of the QP in post-Brexit Britain has become increasingly complicated. This is primarily due to the complex supply chains that are now in place and the variation in processes depending on where a product has come from and what type of drug it is. These revisions to clinical legislation are all occurring within the context of a changing pharmaceutical landscape, with different products being tested and different trial structures being implemented.

### Remote sampling in clinical trials

Dr. Neil Spooner (Spooner Bioanalytical Solutions) introduced advances into how remote blood sampling can improve not only the outcomes of clinical trials but also the patient experience. Traditional forms of blood sampling (venous phlebotomy) collect several milliliters of blood, most of which is not required for scientific procedures and the excess ends up being discarded. Modern, commercially available technologies can overcome these unethical procedures and simultaneously benefit clinical trials.

Patient-centric sampling (PCS) is a remote blood collection process that is at the forefront of clinical innovation and came to particular prominence during the COVID-19 pandemic. This technology, often termed micro sampling, involves collecting smaller volumes of blood, in locations away from clinical centers, from areas of the body with fewer pain receptors. Areas such as the upper arm or middle back are well vascularized and produce high-quality blood samples. This technology can sample as little as 10 µL of blood which can then be dried for easier and cheaper transportation. Crucially, this process can be performed by the patient in the comfort of their own home, thus drastically reducing the impact of the procedure while never compromising the integrity of the sample collected.

In addition to focusing more attentively on the needs of the patient, PCS also tackles several longstanding challenges associated with clinical trials. By introducing remote sampling, studies are no longer limited by the catchment areas of local hospitals and study centers. In many clinical trials, patients from rural locations are often neglected; if patients are not close to a study center, then they cannot be enrolled. This focus on patient convenience can also benefit a number of vulnerable populations, such as pediatric, geriatric, and oncology patients or those with rare diseases. The benefits of remote sampling allow study sponsors to structure more ambitious trials to include a greater variety of patients. Using PCS can also address problems with recruitment and retention. In essence, patients may be more willing to participate if they do not need to travel so often to a site. Instead, patients can conveniently collect the sample at home and mail it to the trial site, a method successfully implemented during the COVID-19 pandemic.

Blood sampling for pharmacokinetics (PK) is also amenable to PCS. Clinical trial endpoints are often limited by the maximum blood collection volume allowed to be drawn from a patient. Reducing the volume sampled can allow for more extensive—and more comprehensive—PK sampling and therefore modelling, leading to more ambitious trial endpoints and a better understanding of the studied disease. However, it should be acknowledged that PCS does not lend itself so readily to collecting a full PK profile, which can require multiple, and specifically timed, collections over a short period (such as 15 time points in 12 h). Rather, PCS is better suited for time points when patients are at home during later stages of the trial.

Using PCS also has the potential to alleviate some of the costs associated with clinical trials. Patients can be sent home from the clinic sooner if they can collect a sample at home, thus lowering the cost of their hospital stay. By only collecting a small volume of blood that is then dried, shipping costs are drastically reduced compared to transporting larger chilled containers. By having all trial sites use a standardized method of remote sampling, less time, effort, and cost will be required to normalize PK data.

Limitations to PCS include logistical concerns and implementing international regulatory guidelines. Patient anonymity when using a remote blood sample is a significant challenge to incorporating PCS, as is ensuring that samples are being sent or delivered properly, or that they are being stabilized appropriately. By introducing remote sampling, there needs to be consistency in how patients correctly perform all steps of the procedure. Questions have been asked regarding how different international regulators may govern PCS; additionally, further challenges may be introduced for internationally conducted trials. However, the US Food and Drug Administration is leading the way internationally for incorporating PCS in trials, and Dr. Spooner hoped this would encourage the same across other regulatory institutions.

Dr. Spooner concluded that PCS is an exciting innovation, but one which must be fully embraced by the field before the benefits are experienced. Concerns around Regulatory acceptance pose an immediate barrier to this technology being used in clinical trials, and logistical challenges must be addressed before PCS can be efficiently used to help studies improve patient recruitment and welfare.

## Afternoon session

### Keynote speech: a retrospective of 30+ years in early clinical developments

The keynote speaker for the conference was Prof. Liz Allen (IQVIA), who also shared the experiences and opinions of Prof. Tim Mant (IQVIA), who presented a retrospective of significant changes in early clinical development over the past 40 years. Clinical trials have changed drastically since the 1970s. Originally, Phase I studies were not regarded as therapeutic trials, therefore, not subject to regulatory or ethical approval. With the exception of some academic institutions, trials were conducted by privately owned industry units. Since then, numerous regulations and innovations have helped make early phase clinical trials safer and more efficient.

The Association of the British Pharmaceutical Industry first issued guidelines for the conduct of Phase I studies in 1970 (Association of the British Pharmaceutical Industry, 1970), and subsequently revised in 1977 and 1988 (Association of the British Pharmaceutical Industry, 1977; Association of the British Pharmaceutical Industry, 1988a; Association of the British Pharmaceutical Industry, 1988b). It was only in 1996 that the International Council for Harmonisation published the E6 guidelines on Good Clinical Practice (Dixon, 1998), and yet these were still not legally mandated regulations. Phase I trials in the UK only became ‘regulated’ with the implementation of the European Directive in May 2004 (published in 2001) (Clinical Trials Directive, 2001).

Since 2000, Phase I trials have had to adapt to the increasingly innovative nature of medicinal products. Chemical entities started to include small biologics, new proteins, and monoclonal antibodies. Increasingly, cohorts of the target population were included for proof-of-mechanism or proof-of-concept, focusing on the type of molecule and disease indication. Research programs were becoming more complex, but they fell under a single ‘umbrella’ protocol, with use of appropriate biomarkers for safety and pharmacodynamics.

Alongside the growing innovation of Phase I trials, significant safety incidents pressured the industry to critically evaluate the welfare of trial participants. The first recorded serious safety incident in a trial occurred in August 1984, with the death of a ‘healthy’ study participant (Dunne, 1984). Investigation found that interaction between the medicinal product and the individual’s concurrent medication led to their death. This incident highlighted a key consideration of medical record data and its need to be validated. Another important event was the death of a medical student volunteer in 1985 (British Medical Journal, 1985). This individual volunteered to take part in the study only 4 months after finishing another study. The screening visit helped diagnose the volunteer with aplastic anemia, which eventually resulted in their death. This event brought to attention the frequency with which volunteers should participate in trials. The Royal College of Physicians subsequently published a guideline on best practice for the conduct of clinical trials in healthy volunteers (Royal College of Physicians, 1986), The Association of Independent Clinical Research Contractors produced similar gudance that also included instructions on the collecting of data around any reported serious adverse events in early phase studies (Association of Independent Clinical Research Contractors, 1989). These days, ethical approval will not be granted if a patient is concurrently participating in another trial, or if an individual has taken part in a trial recently; this exclusion criteria is also part of all trial protocols.

More recent incidents ushered in more extensive safety requirements. Consequences of the TGN1412 episode in 2006, where six patients experienced a cytokine storm, introduced the minimum anticipated biological effect considerations and saw the EMA Committee for Medicinal Products for Human Use publish the ‘Guideline on strategies to identify and mitigate risks for first-in-human clinical trials with investigational medicinal products’ in 2007 (European Medicines Agency, 2007). These guidelines were later expanded to also focus on multiple-ascending dose studies (European Medicines Agency, 2017). Safety incidents, such as those during the TGN1412 trial, brought into stark focus the concept that complex drugs and molecules were now being developed and tested; therefore, extensive background knowledge on how they interact with signaling and cascade pathways within the body and understanding of how to prescribe rescue medication was essential to enable trial approval. Established in 2010, the MHRA Phase One Voluntary Accreditation Scheme dictated that investigators should have relevant clinical experience in running Phase I trials, such as hospital based clinical pharmacologists, and that postgraduate qualifications, such as the Diploma in Human Pharmacology and the MSc in Clinical Pharmacology, or equivalent, were required for investigators to run clinical trials.

Recently revised EMA guidelines on early phase studies in 2017 have emphasized more rigorous preclinical pharmacology evaluations (European Medicines Agency, 2017). The utilization of emerging PK and pharmacodynamic data is required to inform dosing strategy. Furthermore, maximum tolerated dose objectives are no longer acceptable in healthy volunteer study designs. New legislation has been introduced in the UK as a result of Brexit, including the Medicines for Human Use (Clinical Trials) (Amendment; EU Exit) Regulations 2019 and the Medicines and Medical Devices Act (2021) (UK Government legislation, 2019; UK Government legislation, 2021). Due to these regulations, Prof. Allen argued that Phase I studies are now incredibly safe. The incidence of a serious adverse event in a non-geriatric patient is approximately 0.3% (Sibille et al., 2006), and the risk of death occurring in a healthy volunteer is approximately 1 in 1,000,000 (adapted from Sibille et al., 2006 with updated death report in 2016).

Prof. Allen concluded that innovations are always being sought to improve safety and mitigate risks. Following the COVID-19 pandemic, there is increased interest and participation from the public in clinical research—enthusiasm that should not be wasted. The UK remains world leader in Phase I studies due to its record and reputation for scientific innovation and development. This global recognition is also due to the expertise, rigor, and clear communication of the MHRA, all of which are attributes particularly appreciated by US sponsors. Nonetheless, as our understanding of the pathological basis of disease improves, so will modern therapeutic technologies. We have to stay vigilant to better understand not only the challenges we may encounter, but also the risks that may come in the future.

### Conducting challenge trials in healthy volunteers and patients–lessons learned

Dr. Ellis spoke about the processes involved when chairing the ethical committee presiding over the COVID-19 challenge trials and what lessons were learnt.

In May 2022, the World Health Organization issued guidance on challenge trials relating specifically to COVID-19. A challenge study consists of administering a substance to an individual where the outcomes are unknown: Either the individual has a disease, or they are incubated with one. In the case of COVID-19, challenge studies were conducted to learn more about how the virus functioned and begin vaccine efforts. The UK was the first country to run these studies and considering the unique nature of the ethical approval required, Dr. Ellis was approached to chair the committee. The structure of the committee did not follow a traditional format; rather, a greater proportion of professional representatives were present, with only 25% of the committee being lay individuals, compared to the 33% normally seen in ethics committees. As such, Dr. Ellis considered the approval process to be limited by the under-representation of the opinions of the general public.

The first topic of consideration for the COVID-19 challenge committee was safety. Due to the very nature of the trial, safety could not be guaranteed to the same extent as a normal clinical trial. Discussions therefore progressed to the confidence of investigators that the substance being prescribed was ‘suitable.’ Likewise, confidence levels needed to be established as similarly suitable for the efficacy of any rescue medications. The COVID-19 challenge ethics debate had a higher degree of complexity that required longer deliberations, which Dr. Ellis attributed to a lack of understanding by the committee members of potential issues that may arise during the course of the planned investigations.

One key procedure implemented by the challenge committee was to support the ethical consent procedures of the study. It was suggested that after initial consent was given by a volunteer, they would be asked a second time immediately before starting the trial if they were happy to proceed. It was to be made clear to the individual that there would be no consequences if they wanted to back out.

Another topic of contention was the demographic of volunteers. The QCOVID Score is an algorithm used to assess mortality rates from COVID-19, and was included in the study’s methodology (Clift et al., 2020). However, this score acknowledges that individuals from ethnic minority groups are more susceptible to COVID-19, and therefore, the investigators decided that including any such individuals could produce unreliable data. Dr. Ellis conceded that the demographic of individuals in the study was misrepresentative of the general population, again undermining the procedures of the ethical approval process. Other aspects of the pandemic that were not appropriately captured in the challenge study were pathologies from the long-term effects of COVID-19 or the dangers of its variants, such as the delta or omicron variants. However, at the time, these factors were not fully established and did not merit discussion.

In summary, Dr. Ellis stressed that the COVID-19 challenge committee meetings involved topics of discussion that were unprecedented in modern day therapeutic trials. As such, the processes and outcomes of the meeting perhaps did not achieve the traditional standards of regulatory ethical approvals.

### Early phase principal investigator accreditation and certification in the UK

Dr. Ulrike Lorch (Richmond Pharmacology) opened the discussion presenting work done as Director of Human Pharmacology of the Faculty of Pharmaceutical Medicine alongside a stakeholder group. This group consists of Principal Investigators (PIs) with academic, commercial, and National Health Service backgrounds; specialty trainees in Clinical Pharmacology, Therapeutics, and Pharmaceutical Medicine Specialty Training; and MHRA and Health Research Authority representatives. Principal Investigator accreditation first arose in the UK following the outcomes of the TGN1412 incident in 2006. The 2006 Duff Report, and the subsequent 2008 MHRA Phase One Accreditation Scheme, mandate d that PIs need suitable qualifications when conducting early phase trials, and that study centers need similar accreditation for these types of higher-risk investigations (Expert Scientific Group, 2006; UK.GOV, 2023).

Since 2006, the scope of work of early phase PIs has evolved beyond traditional clinical pharmacology studies, with more complex therapeutic technologies being explored in diverse research environments. Dr. Lorch proposed that an updated PI accreditation and certification scheme is necessary to accommodate the innovations seen in early phase trials.

The proposed update consists of three main steps. The first step has been completed: To ensure that the accreditation and certification given is relevant to the scope and nature of trials being conducted. There is currently a large variety of early phase trials being run in the UK, with a diverse range of PIs overseeing them. Therefore, the proposed training and accreditation programs must reflect the differing backgrounds and workplaces. The curriculum of core capabilities needs to focus on innovative study designs, therapies, and patient populations, with emphasis on medical oversight and clinical risk management. The stakeholder group have produced a curriculum of core capabilities all early Phase PIs require, irrespective of their scope of work. Identified capabilities were categorized into two streams: Over-arching themes (applied human pharmacology capabilities for modern PIs) and clinical trial journey (from study concept to publication). Proposed capability levels would reflect those employed by the Pharmaceutical Medicine Specialty Training curricula. Individual capabilities can be mapped to existing specialty trainings, such as the Pharmaceutical Medicine Specialty Training and Clinical Pharmacology and Therapeutics.

The second step of the proposal is to make the accreditation and certification of PIs readily accessible. Accessibility for the wide scope of PI duties can be promoted by encouraging collaboration with other experts to fill potential ‘capability’ gaps. Accessibility can also be promoted by using training and assessment systems or processes that are used by all doctors within current appraisal systems (including revalidation and annual reviews that comply with General Medical Council processes). Organizing an expert group of appraisers and educational supervisors would facilitate review of PIs’ annual appraisal portfolios in workplaces where higher capability levels can currently not be assessed.

The final step in the proposal is formal certification of the ‘capabilities’ PIs have achieved over the appraisal year. Certification acknowledges achievements and inspires confidence and trust from patients, regulators, collaborators, and sponsors.

There could be two pathways for early phase PI certification, which would run in parallel. The first pathway could be via a formal General Medical Council credential. This would likely be attractive for doctors currently in specialty training, or those with a large proportion of PI work. The second could be a more flexible system, where PIs could maintain certification on an annual basis tailored to their scope of work. This would likely be more attractive for clinical and academic specialist with a smaller proportion of PI duties.

Following Dr. Lorch’s talk, Dr. Kirsty Wydenbach (Weatherden) discussed the benefits of introducing regulatory education into the training schedule of PIs. Understanding the processes of clinical trials from a regulatory perspective can be of huge benefit to the everyday role of a PI. Emphasis would be placed on regulatory aspects such as reviewing and re-evaluating the risk-benefit balance with an understanding of why these steps are important. By appreciating regulatory legislation, PIs can have a rounded appreciation of the clinical trial process and, crucially, patient safety. Furthermore, this training would enable PIs to think more like a regulator. This insight would benefit all aspects of a trial, from developing the study design, through to drafting the protocol, releasing development safety update reports, any post-trial publications, and beyond. Principal Investigators would also be better placed to converse effectively with regulatory authorities.

Dr. Lorch and Dr. Wydenbach explained that the concept is still in development. Next steps would include involving key UK institutions, such as the General Medical Council, MHRA, Health Research Authority, and British Pharmacological Society. With these bodies on board, this proposal for improved PI training and accreditation can target most clinical trials and also be applied to most medical specialties.

### Market considerations and early phase investment

The Deloitte Centre for Health Solutions performs an annual analysis of the top 20 pharmaceutical companies in the world to establish how they can improve the performance of their clinical research and development processes (Deloitte Centre for Health Solutions, 2021). Dr. Naveed Panjwani (Deloitte) explained this report and discussed the return on investments made in clinical research and development and how an in-depth analysis identifies areas of innovation.

The annual Deloitte report collates data from assets of the leading pharmaceutical companies gathered by GlobalData [https://www.globaldata.com] to calculate the internal rate of return (IRR): A measure of the return on investment for shareholders in a company. The IRR is calculated by first assessing the pharmaceutical assets deemed likely to provide medical value or financial revenue to the company. These assets include Phase II products with a breakthrough designation, Phase III products, and products that have undergone database lock and are awaiting regulatory approval. The secondary input of data to calculate the IRR is an estimation of future sales (20+ years). This is a consensus of what revenue analysts typically expect the products to earn. With these figures, companies estimate costs for all phases of drug development. Mean IRR tracks investment in pharmaceutical companies over the past 10 years to provide insight into the direction of the pharmaceutical industry.

When first calculated in 2010, the industry IRR was returning approximately 10%. However, this value has since fallen, and in the past 4 years, it has ranged from 1.5% to 3.6%. There was a rise in 2020 and 2021, owing to COVID-19 therapeutics and vaccines. However, when adjusted for these emergency authorizations, productivity during these years adhered to the downward trend and reached an IRR of 3.2%. Dr. Panjwani revealed that for 2021–2022, IRR calculations are expected to be even lower. Ultimately, this indicates that pharmaceutical companies are not returning value above the cost of the capital and that the drug development process is inefficient and unsustainable.

Productivity of the drug development process can be analyzed at three levels: Number of late-stage pipeline assets; forecast average peak sales per asset; and research and development cost to bring an asset to market. The number of outputs from the pharmaceutical pipeline has remained relatively level over the past decade, suggesting that despite the large scope and size of top pharmaceutical companies, there is no great innovation in how pipelines process assets. Research leads acknowledge the costs of many terminated assets, and these costs unfavorably balance out the pipeline. Conversely, regulatory authorities must also initiate movement to not limit IMP progression. Dr. Panjwani expressed it is likely that such constraints to the pharmaceutical pipeline are affecting the productivity of clinical organizations.

Another aspect of productivity reflects peak sales per asset. In general, ‘blockbuster’ drugs are no longer achievable. Previous drugs generated sales of up to $10 billion annual revenue for some companies, whereas most current products yield in the range of $400–$500 million. This effect is likely a consequence of more specific targeting of disease indications, resulting in fewer patients eligible to treatment. In addition, the report and recommendations of the 2019 Ways and Means Committee in the US resulted in pharmaceuticals being priced more reasonably, with greater emphasis on economic value and quality of life benefits. These drugs undergo greater scrutiny before reimbursement, potentially limiting overall sales.

Finally, productivity is reflected in the cost of bringing a drug to market, including the cost of attritional and terminated projects. During 2019, the peak average cost was $2.4 billion per drug. From a consulting perspective, the cost of bringing a drug to market is the challenge that offers the most opportunity for value innovation and change in the pharmaceutical industry. However, when considering the decreasing revenue and number of stagnant assets in the therapeutic pipeline, the increased costs are also the most damaging level of productivity facing the pharmaceutical industry.

Dr. Panjwani explored where new therapies originate and what modalities are primarily in development. For big pharmaceutical companies, 50% of programs have traditionally originated in-house and 50% would be acquired from smaller biotechnologies. However, in recent years this balance has shifted, where the majority (∼70%) of projects are originating from external ventures such as acquisitions or co-development agreements. These trends indicate that smaller companies are driving innovation, but these assets are ultimately bought by larger firms along with the associated study teams. Of these projects, the modalities currently in development are dominated by antibody technology, making up 80% of all research products, followed by traditional small molecules. An increase in the number of oligonucleotide and nucleic acid-based medicines indicate a shift towards cell and gene therapies. Overall, current trends are showing that innovation is being driven by smaller companies with a rise in cell and gene therapies.

Dr. Panjwani discussed how pharmaceutical companies have been trying to increase their productivity by speeding up the clinical trials process. This aims at reducing spend; the greatest cost in drug development is human time and effort. However, in reality, trials are becoming more and more complex, and taking longer to deliver compared to 5–10 years ago. Considering the complexities associated with new and innovative technologies, companies appear to focus their efforts on developing new techniques for patient recruitment and better retention, faster study start-up, or simpler study protocols.

### Interactive session: working with the MHRA

Current interactions between the UK clinical trials community and the MHRA appear to be operating sub-optimally. The departure of many experienced inspectors and assessors has resulted in a severe impact on available resources. Consequently, regulatory processes have been taking longer, impacting the efficiency of clinical studies across the United Kingdom. Dr. Keith Berelowitz (Richmond Pharmacology) led a discussion session to explore ways the clinical community can work together to improve the current landscape and ensure UK Phase I units continue to play a vital role in global research.

**FIGURE 2:**
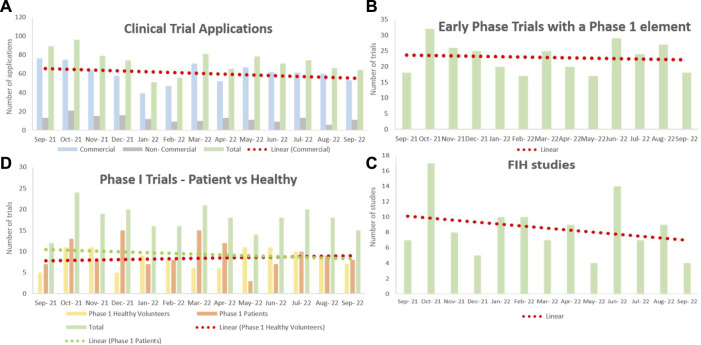
The number of clinical trial applications to the MHRA between September 2021 and September 2022. Data collected from Phase I units across the United Kingdom. Normalized by removing COVID-19 studies, and linear regression models fitted. **(A)** A downward trend was observed for the number of CTA applications in general. The majority of applications were commercial. **(B)** The number of applications of early phase trials with a Phase I element remained constant throughout the year. **(C)** There were slightly fewer applications for Phase I patient trials, whereas the number of applications for Phase I healthy trials steadily increased throughout the year. **(D)** The number of FIH study applications has notably declined over the past year. CTA = clinical trial authorization; FIH = first-in-human; MHRA = Medicines and Healthcare products Regulatory Agency.

Dr. Berelowitz first provided context on the current landscape of early phase clinical trials by providing data on CTA applications processed between September 2021 and September 2022 (Figure 2). A downward trend was observed for the number of CTA applications in general as well as for Phase I patient trials and first-in-human trials. Dr. Berelowitz presented the results of a pre-meeting survey sent to Phase I units across the UK regarding recent interactions with the MHRA. The results overwhelmingly indicated that CTA applications are taking longer to be approved. Where a first response to an application used to occur within 15 days, two-thirds of units recorded that 50% of their 2022 applications had a 30-day wait time before an initial MHRA response. Furthermore, the majority of sites experienced a wait time of over 30 days to receive approval after a grounds for non-acceptance letter. Overall, these data appear to confirm that the MHRA is currently experiencing a significant period of disruption that is impacting negatively on regulatory interactions with the clinical trials community.

Despite concerns over the apparent longer interactions and responses from the MHRA, sponsors were more concerned that the MHRA responses had become less predictable. Sponsors prefer agencies that are innovative, informed, and willing to listen to the community, but there must also be consistency. The discussion therefore centered mainly on what the Phase I community can do to improve interactions, and what can be done to assist regulators, not only in regard to early phase clinical trials but for all components of the industry.

Dr. Berelowitz stressed that the results of the pre-meeting survey and the comments collected from the discussion session were being conducted in a collegiate and collaborative fashion with the aim of serving the MHRA. The MHRA’s executive board were eager to hear feedback from the community. The current state of clinical trials in the EU presents opportunities for UK research organizations to exploit the challenges seen with the Clinical Trials Information System that has discouraged pharmaceutical and biotechnology companies.

Following the introduction, Dr. Berelowitz opened up discussion to the floor to establish a position of the clinical trials community and to determine the correct direction for future change within the MHRA.

A key takeaway from the session was that the central issue appears to be one of resources. Simply, there are currently not enough personnel to address the needs of the clinical community, and re-allocating resources is not a sustainable solution. In the Clinical Trials Unit of the MHRA there is currently only one non-clinical assessor, four medical assessors, and four pharmaceutical assessors. Not only is this insufficient to deal with current demand, there are few resources to perform the necessary training to bring on new team members, and even if there were, it is likely to take approximately 2 years for benefits to emerge. As such, MHRA regulators are having to prioritize their workload, which is done by categorizing work by the action needed. The clinical community can assist in this by clarifying what is required, thus allowing for regulatory interactions to be dealt with and prioritized more efficiently by the MHRA. In addition, communicating with the regulators before an application can help prevent any unnecessary delays during the regulatory process. Dr. Berelowitz summarized that while resource constraints are the fundamental issue, this should not be mistaken for inadequate or poor finances. The MHRA is resource light, and simply generating more money will not necessarily solve the issues.

As a result of the resource challenges, the clinical community is experiencing inconsistencies with how approvals and interactions are being conducted. One audience member recounted concerns over chemistry, manufacturing, and controls review. Another example included stability data in the method section of a CTA not being thoroughly checked. These are important issues considering sponsors have increasingly complex studies and tight deadlines and increasing the risk of below standard applications. Overall, it was concluded that a lack of experience and resources at the MHRA will result in a slower approval rate, inappropriate trials being run, and an impact on the competitiveness of the UK in the Phase I trial space.

The primary action established during the discussion session was for the formation of a stakeholder group that could connect the opinions of the various parties involved and present a unified argument to the relevant authorities. Although the MHRA is an independent agency, it is still part of the civil service. The challenges faced by the MHRA will impact on the competitiveness of UK’s pharmaceutical industry. Therefore, informing the government of these concerning issues is an appropriate action to take. An approach that addresses local members of parliament as well as those higher up in the House of Lords can help indicate that the problems with the MHRA will have a widespread impact on the pharmaceutical industry. Dr. Berelowitz summarized that the message from this stakeholder group would need to be concise and well communicated to the government and the MHRA. The expected output from this communication should be to solve problems with timelines and with how applications are received.

### What is the cost of a day in clinical development?

Dr. Graham Wylie (Medical Research Network [MRN]) discussed an alternative approach to analyzing the value of clinical trials and explored how adoption of decentralizing trials methodology can reduce costs in clinical drug development and increase patient engagement.

Two main aspects must be considered when determining the costs of clinical trials. First, how to measure value, and second, the volume to be measured. Dr. Wylie argued that these two questions must be considered in terms of the exercises ultimate goal, delivering effective treatments to patients as quickly and effectively as possible.

Establishing the cost of bringing a drug to market is best achieved by focusing on the turnover of single-product companies, this way the attritional costs of failed drugs are not included. With this approach, the average cost to develop a drug can be calculated as $200–$400 million that tends to be expended over the course of approximately 7 years. This simplifies to $29 million per year or $112,000 per day. When taking the approximate number of IMPs in development at any one time (approximately 7000), reducing clinical trials by 1 day globally can save the industry $780 million. This calculation measures value from a pharmaco-centric perspective, where clinical development is measured by how little costs can be expended during the drug development phase, and then to generate the potential maximum profit during the marketing phase.

The other measurement of importance in clinical development is people, in particular, patient days. Using a Parkinson’s drug from a client of MRN as an example, Dr. Wylie calculated that a 1-day reduction leads to approximately 6650 more patient days. Considering 90% of products do not make it to market, a 1-day reduction for the 700 marketed drugs would result in ∼4.7 million more patient days.

Thus, calculating the costs of a single day in drug development can be relatively simple, introducing the opportunity to determine where and how the reductions can be made is the challenge. Decentralized clinical trials aim to reduce the need for patients to travel to central trial sites and provide opportunities to improve several costly aspects of clinical drug development. Instead of targeting the efficiency of trial sites, where all the operational and clinical work takes place, a focus on regulatory issues or patient engagement can be of more financial benefit for pharmaceutical companies.

Estimates suggest that clinical trial sites currently only approach ∼30% of all available patients for a certain study, indicating a fundamental inefficiency. There are three challenges that need to be addressed to improve patient engagement. The first challenge is that trial sites are often too far away from many of the eligible patients. Most patients enrolled to a trial live within 10 miles of the site, those outside of this catchment area often go unstudied. The second challenge is time. Trial visits can only take place during the time allocated for them by the trial site. Often, these timings do not account for participants’ daily activities, thereby affecting their ability to visit the study center. Dr. Wylie explained how 80% of patients assessed by MRN were unable to attend visits during standard work hours. The final challenge focuses on resources. For every 10 patients that are screened by a study center, only 0.8 proceed to randomization. This slows down the recruitment process as centers are limited by how many participants they can screen at any one time.

Decentralized clinical trials address all three of these issues. Patients are given the freedom to be seen where they want, when they want, and nurses are transferred to trial sites to strengthen resources and allow for a greater number of participants to be screened simultaneously. As such, more patients are successfully recruited to the clinical trial in a shorter period of time. On average, MRN improved recruitment by 50% at their clients’ sites, reducing the screening period and overall length of the trial by an average of 5 months. The same Parkinson’s trial saw the recruitment period shorten from 18 months to 10 months, equating to $225 million in new revenue when the drug eventually hits the market and 4.9 million patient days.

In concluding his presentation, Dr. Wylie noted that decentralized clinical trials are a valuable alternative to traditional methodologies not only for the significant savings that can be made, but also for the benefits to patient welfare. With a clinical trial shortened by ∼5 months, and if this was applied to all products currently in development, it would generate $17 billion in revenue and 55 million patient days.

## Close and conclusion

The 2022 AHPPI conference covered a broad range of topics regarding the future of innovation and change within early phase clinical drug development. The impacts of Brexit and the COVID-19 pandemic on the UK pharmaceutical markets and clinical practices were running themes throughout the day. While the productivity of the top pharmaceutical companies was analyzed and the impacts of these trends discussed, emphasis was primarily on how the patient perspective can be improved via such innovations as remote sampling and decentralized clinical trials. An engaging discussion broached the topic of current issues within the MHRA and suggested actions included the formation of a stakeholder group to present a unified argument to the UK government. Debaters found common ground regarding the ethics of whether Phase I trials should be conducted the same in oncology patients as healthy subjects. The keynote speaker contextualized future directions for change by providing a retrospective on 40 years in early drug development. Further intriguing topics included the challenges associated with gene therapies, the role of the QP post-Brexit, and proposals for the future of PI certification-related training in the UK. Insight was also provided on the COVID-19 challenge trials, and what lessons were learnt from the unfamiliar ethical committee. In closing the meeting, AHPPI Chairman, Dr. Hardman, summarized the position of the UK following the implementation of the Clinical Trials Regulation and the Clinical Trials Information System, and the unique opportunities for innovation.

## References

[B1] Association of Independent Clinical Research Contractors (1989). Guidelines for research ethics committees. Oakland, CA, USA: AICRC.

[B2] Association of the British Pharmaceutical Industry (1970). The report on medical experiments in staff volunteers. Oakland, CA, USA: AICRC

[B3] Association of the British Pharmaceutical Industry (1977). Guidelines for preclinical and clinical testing of new medicinal products. Oakland, CA, USA: AICRC.

[B4] Association of the British Pharmaceutical Industry (1988a). Guidelines for facilities for non-patient volunteer studies. Oakland, CA, USA: AICRC.

[B5] Association of the British Pharmaceutical Industry (1988b). Guidelines for medical experiments in non-patient volunteers. Oakland, CA, USA: AICRC.

[B6] Association of the British Pharmaceutical Industry (2021). Clinical research in the UK: an opportunity for growth. Available at: https://www.abpi.org.uk/media/g0anpn5o/abpi_clinical-trials-report-2021.pdf.

[B7] British Medical Journal (1985). Death of a volunteer. Br. Med. J. Clin. Res. Ed. 290 (6479), 1369–1370. PMC1415641 3158369

[B8] Chihara C. Lin R. Flowers C. R. Finnigan S. R. Cordes L. M. Fukada Y. (2022). Early drug development in solid tumours: analysis of National Cancer Institute-sponsored phase 1 trials. Lancet 400 (10351), 512–521. 10.1016/S0140-6736(22)01390-3 35964611 PMC9477645

[B9] Clift A. K. Coupland C. A. C. Keogh R. H. Diaz-Ordaz K. Williamson E. Harrison E. M. (2020). Living risk prediction algorithm (QCOVID) for risk of hospital admission and mortality from coronavirus 19 in adults: national derivation and validation cohort study. BMJ 371, m3731. 10.1136/bmj.m3731 33082154 PMC7574532

[B10] Clinical Trials Regulation (2014). Regulation (EU) No 536/2014. Regulation (EU) No 536/2014 of the European Parliament and of the Council of 16 April 2014 on clinical trials on medicinal products for human use, and repealing Directive 2001/20/EC Text with EEA relevance. [Accessed 31/01/2023]. Available at: https://eur-lex.europa.eu/legal-content/EN/TXT/?uri=celex%3A32014R0536.

[B11] Clinical Trials Directive (2001). Directive 2001/20/Ec of the European Parliament and of the Council Of 4 April 2001 on The Approximation Of The Laws, Regulations and Administrative Provisions of The Member States Relating to The Implementation of Good Clinical Practice in the Conduct of Clinical Trials on Medicinal Products For Human Use. Amsterdam, Netherlands. Clinical Trials Directive. 16276663

[B12] Clinical Trials Information System (2023). EU Clinical Trials. [Accessed 31/01/2023]. Available at: https://euclinicaltrials.eu/.

[B13] Deloitte Centre for Health Solutions (2021). Nurturing growth. Measuring the return from pharmaceutical innovation 2021. Available at: https://www2.deloitte.com/content/dam/Deloitte/uk/Documents/life-sciences-health-care/Measuring-the-return-of-pharmaceutical-innovation-2021-Deloitte.pdf.

[B14] Dixon J. R. (1998). The international conference on harmonization Good clinical practice guideline. Qual. Assur 6 (2), 65–74. 10.1080/105294199277860 10386329

[B15] Dunne D. (1984). The death of niall rush – an experiment in james street. *Magill.ie*. [Accessed 31/01/2023]. Available at: https://magill.ie/archive/death-niall-rush-experiment-james-street.

[B16] European Commission (2015). EU guidelines for Good manufacturing practice for medicinal products for human and veterinary use. Annex 16: certification by a qualified person and batch release. Available at: https://www.gmp-compliance.org/files/guidemgr/v4_an16_201510_en.pdf.

[B17] European Commission (2022). The rules governing medicinal products in the European union volume 4 EU guidelines for Good manufacturing practice for medicinal products for human and veterinary use Annex 21: importation of medicinal products. Available at: https://health.ec.europa.eu/system/files/2022-03/vol4_annex21_en.pdf.

[B18] European Medicines Agency (2007). Guideline on strategies to identify and mitigate risks for first-in human clinical trials with investigational medicinal products. Amsterdam, Netherlands. European Medicines Agency.

[B19] European Medicines Agency (2017). Guideline on strategies to identify and mitigate risks for first-in human clinical trials with investigational medicinal products – revision 1. Amsterdam, Netherlands. European Medicines Agenc

[B20] European Medicines Agency (2020). Libmeldy. [Accessed 31/01/2023]. Available at: https://www.ema.europa.eu/en/medicines/human/EPAR/libmeldy.

[B21] Expert Scientific Group (2006). Expert scientific group on phase one clinical trials-final report. Available at: https://hansard.parliament.uk/Commons/2006-12-07/debates/06120763000018/Phase1ClinicalTrials(ExpertScientificGroup).

[B22] Faculty of Pharmaceutical Medicine (2021). Curriculum for pharmaceutical medicine specialty training – implementation august 2021. Available at: https://www.fpm.org.uk/wp-content/uploads/2022/04/20210716-PharmMed-Curriculum-2021_final-1.0-2.pdf.

[B23] Fumagalli F. Calbi V. Sora M. G. N. Sessa M. Baldoli C. Rancoita P. M. V. (2022). Lentiviral haematopoietic stem-cell gene therapy for early-onset metachromatic leukodystrophy: long-term results from a non-randomised, open-label, phase 1/2 trial and expanded access. Lancet 399 (10322), 372–383. 10.1016/S0140-6736(21)02017-1 35065785 PMC8795071

[B24] GlobalData (2023). Available at. https://www.globaldata.com/ (Accessed 01/02/2023).

[B25] National Institute of (2023). Neurological disorders and Stroke. [Accessed 31/01/2023]. Available at: https://www.ninds.nih.gov/health-information/disorders/metachromatic-leukodystrophy.

[B26] Royal College of Physicians (1986). Research on healthy volunteers. A report of the royal College of physicians. J. R. Coll. Physicians Lond 20 (4), 243–257. 3772845 PMC5371045

[B27] Sibille M. Donazzolo Y. Lecoz F. Krupka E. (2006). After the London tragedy, is it still possible to consider Phase I safe? Br. J. Clin. Pharmacol. 62 (4), 502–503. 10.1111/j.1365-2125.2006.02740.x 16817849 PMC1885163

[B28] Uk.GOV (2023). MHRA phase I accreditation scheme. Available at: https://www.gov.uk/guidance/mhra-phase-i-accreditation-scheme#full-publication-update-history.

[B29] UK Government legislation (2004). The medicines for human use (clinical trials) regulations 2004. Available at: https://www.legislation.gov.uk/uksi/2004/1031/made. 15812991

[B30] UK Government legislation (2019). The medicines for human use (clinical trials) (amendment) (EU Exit) regulations 2019. Available at: https://www.legislation.gov.uk/uksi/2019/744/contents/made.

[B31] UK Government legislation (2021). Medicines and medical devices Act 2021. Available at: https://www.legislation.gov.uk/ukpga/2021/3/enacted.

[B32] World Health Organization (2022). Cancer. [Accessed 17/02/2023]. Available at: https://www.who.int/news-room/fact-sheets/detail/cancer.

